# Smooth muscle AKG/OXGR1 signaling regulates epididymal fluid acid–base balance and sperm maturation

**DOI:** 10.1093/lifemeta/loac012

**Published:** 2022-07-14

**Authors:** Chang Xu, Yexian Yuan, Cha Zhang, Yuchuan Zhou, Jinping Yang, Huadong Yi, Ishwari Gyawali, Jingyi Lu, Sile Guo, Yunru Ji, Chengquan Tan, Songbo Wang, Yongliang Zhang, Qingyan Jiang, Gang Shu

**Affiliations:** Guangdong Laboratory for Lingnan Modern Agriculture, National Engineering Research Center for Breeding Swine Industry and Guangdong Province Key Laboratory of Animal Nutritional Regulation, College of Animal Science, South China Agricultural University, Guangzhou, Guangdong, China; Guangdong Laboratory for Lingnan Modern Agriculture, National Engineering Research Center for Breeding Swine Industry and Guangdong Province Key Laboratory of Animal Nutritional Regulation, College of Animal Science, South China Agricultural University, Guangzhou, Guangdong, China; Guangdong Laboratory for Lingnan Modern Agriculture, National Engineering Research Center for Breeding Swine Industry and Guangdong Province Key Laboratory of Animal Nutritional Regulation, College of Animal Science, South China Agricultural University, Guangzhou, Guangdong, China; International Peace Maternity and Child Health Hospital, Shanghai Key Laboratory of Embryo Original Diseases, School of Medicine, Shanghai Jiao Tong University, Shanghai, China; Guangdong Laboratory for Lingnan Modern Agriculture, National Engineering Research Center for Breeding Swine Industry and Guangdong Province Key Laboratory of Animal Nutritional Regulation, College of Animal Science, South China Agricultural University, Guangzhou, Guangdong, China; State Key Laboratory of Biocontrol, Guangdong Provincial Key Laboratory for Aquatic Economic Animals, School of Life Sciences, Sun Yat-Sen University, Guangzhou, China; Guangdong Laboratory for Lingnan Modern Agriculture, National Engineering Research Center for Breeding Swine Industry and Guangdong Province Key Laboratory of Animal Nutritional Regulation, College of Animal Science, South China Agricultural University, Guangzhou, Guangdong, China; Guangdong Laboratory for Lingnan Modern Agriculture, National Engineering Research Center for Breeding Swine Industry and Guangdong Province Key Laboratory of Animal Nutritional Regulation, College of Animal Science, South China Agricultural University, Guangzhou, Guangdong, China; Guangdong Laboratory for Lingnan Modern Agriculture, National Engineering Research Center for Breeding Swine Industry and Guangdong Province Key Laboratory of Animal Nutritional Regulation, College of Animal Science, South China Agricultural University, Guangzhou, Guangdong, China; Guangdong Laboratory for Lingnan Modern Agriculture, National Engineering Research Center for Breeding Swine Industry and Guangdong Province Key Laboratory of Animal Nutritional Regulation, College of Animal Science, South China Agricultural University, Guangzhou, Guangdong, China; Guangdong Laboratory for Lingnan Modern Agriculture, National Engineering Research Center for Breeding Swine Industry and Guangdong Province Key Laboratory of Animal Nutritional Regulation, College of Animal Science, South China Agricultural University, Guangzhou, Guangdong, China; Guangdong Laboratory for Lingnan Modern Agriculture, National Engineering Research Center for Breeding Swine Industry and Guangdong Province Key Laboratory of Animal Nutritional Regulation, College of Animal Science, South China Agricultural University, Guangzhou, Guangdong, China; Guangdong Laboratory for Lingnan Modern Agriculture, National Engineering Research Center for Breeding Swine Industry and Guangdong Province Key Laboratory of Animal Nutritional Regulation, College of Animal Science, South China Agricultural University, Guangzhou, Guangdong, China; Guangdong Laboratory for Lingnan Modern Agriculture, National Engineering Research Center for Breeding Swine Industry and Guangdong Province Key Laboratory of Animal Nutritional Regulation, College of Animal Science, South China Agricultural University, Guangzhou, Guangdong, China; Guangdong Laboratory for Lingnan Modern Agriculture, National Engineering Research Center for Breeding Swine Industry and Guangdong Province Key Laboratory of Animal Nutritional Regulation, College of Animal Science, South China Agricultural University, Guangzhou, Guangdong, China

**Keywords:** AKG, OXGR1, epididymis, acid–base balance, sperm maturation

## Abstract

Infertility is a global concern attributed to genetic defects, lifestyle, nutrition, and any other factors that affect the local metabolism and niche microenvironment of the reproductive system. 2-Oxoglutarate receptor 1 (OXGR1) is abundantly expressed in the testis; however, its cellular distribution and biological function of OXGR1 in the male reproductive system remain unclear. In the current study, we demonstrated that OXGR1 is primarily expressed in epididymal smooth muscle cells (SMCs). Aging and heat stress significantly reduced OXGR1 expression in the epididymis. Using OXGR1 global knockout and epididymal-specific OXGR1 knockdown models, we revealed that OXGR1 is essential for epididymal sperm maturation and fluid acid–base balance. Supplementation of α-ketoglutaric acid (AKG), the endogenous ligand of OXGR1, effectively reversed epididymal sperm maturation disorders caused by aging and heat stress. Furthermore, *in vitro* studies showed that AKG markedly stimulated the release of instantaneous intracellular calcium from epididymal SMCs and substantially reduced the pH_i_ value in the epididymal SMCs via OXGR1. Mechanistically, we discovered that AKG/OXGR1 considerably increased the expression of Na^+^/HCO_3_^−^ cotransporter (NBCe1) mRNA in the epididymal SMCs, mediated by intracellular calcium signaling. The local AKG/OXGR1 system changed the epididymal fluid pH value and HCO_3_^−^ concentration, thereby regulating sperm maturation via intracellular calcium signaling and NBCe1 mRNA expression. This study for the first time reveals the crucial role of OXGR1 in male fertility and sheds light on the applicability of metabolic intermediates in the nutritional intervention of reproduction.

## Introduction

In modern life, several couples choose to postpone having children due to a variety of socioeconomic factors [1], and infertility in males contributes to approximately 50% of such decisions [2]. Therefore, it has become increasingly important to investigate the effects of aging on the reproductive system. The epididymis is a complex tubular structure in the male reproductive system [3]. After leaving the testis, sperm enter the epididymis, where they undergo a series of structural, biochemical, and functional changes before maturing and becoming fertile [4]. Any error in these processes can lead to infertility in males. Recent studies have found that some external factors such as age [5] and heat stress [6] are often the major causes of male infertility in human and livestock, respectively [7, 8]. These factors can result in infertility in males by interfering with the local metabolism of the testis or epididymis.

2-Oxoglutarate receptor 1 (OXGR1) is a G protein–coupled receptor for α-ketoglutaric acid (AKG), an intermediate metabolite of the tricarboxylic acid (TCA) cycle. OXGR1 is mainly expressed in the testis, kidney, and oviduct [9]. In the kidney, OXGR1 can sense AKG, thereby regulating the reabsorption balance of renal tubules and the formation of angiotensin Ⅱ[10, 11]. Additionally, OXGR1 is also distributed in adrenal medullary cells, and is able to sense the presence of AKG, thus regulating serum epinephrine secretion and fat metabolism [12]. However, the role of OXGR1 in the male reproductive system remains unclear. Previous studies have found that both aging and heat stress model are linked to dramatical metabolism change [13–15]. Therefore, delineating the role of AKG/OXGR1 system in male infertility will provide direct targets for clinical application to improve male reproduction by nutrients.

In the present study, we initially revealed that OXGR1 is expressed in epididymal smooth muscle cells (SMCs), and its levels decline with aging and heat stress. Using OXGR1 global knockout (OXGR1-GKO) and epididymal-specific OXGR1 knockdown (OXGR1-eKD) mouse models, we demonstrated that OXGR1 is essential for epididymal sperm maturation. Epididymal fluid acid–base is balanced by the Na^+^/HCO_3_^−^ cotransporter (NBCe1) in epididymal SMCs. Finally, we characterized the benefits of AKG treatment in epididymal sperm maturation disorders caused by aging and heat stress. Collectively, our results demonstrated that epididymal smooth muscle AKG/OXGR1 signaling plays a crucial role in sperm maturation by regulating the acid–base balance in the tubular fluid. Our findings are crucial to understanding how metabolic intermediates regulate male reproduction.

## Results

### Tissue distribution and localization of OXGR1 in the mouse epididymis in response to aging and heat stress

**Figure 1:**
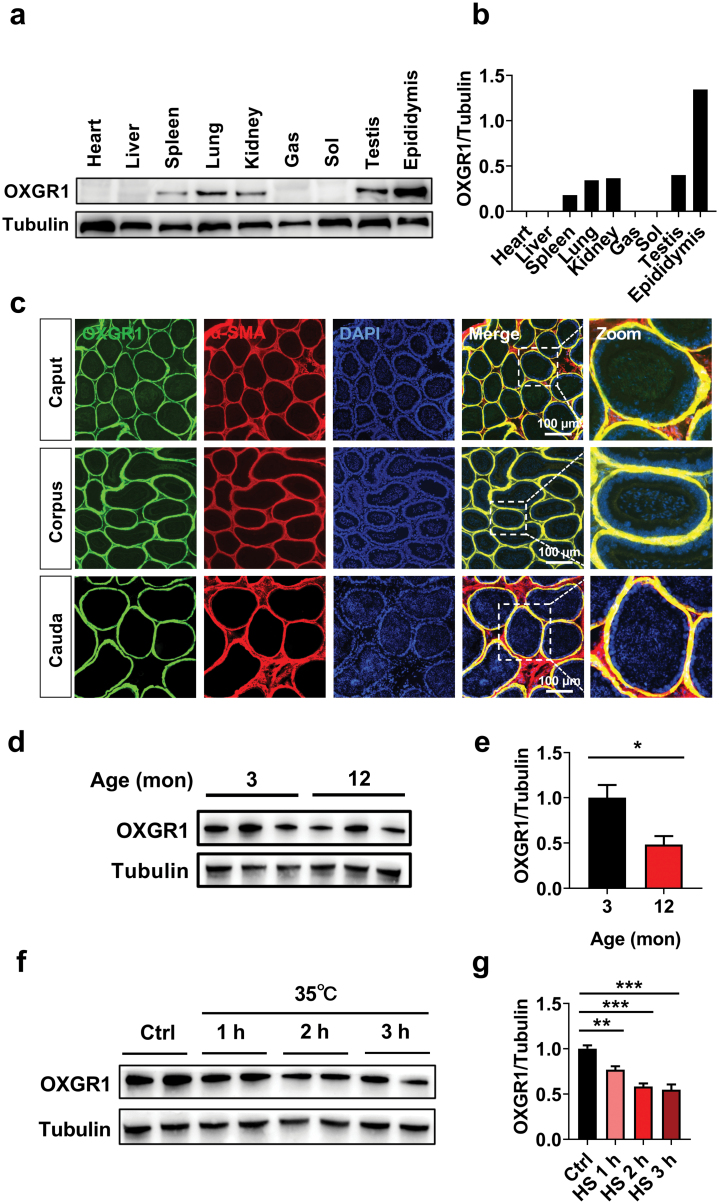
Tissue distribution and localization of OXGR1 in the epididymis in response to aging and heat stress (HS). (a, b) Immunoblots (a) and quantification (b) of OXGR1 protein in heart, liver, spleen, lung, kidney, gastrocnemius (Gas), soleus (Sol), testis and epididymis of mice. (c) Immunofluorescence detection of OXGR1 (green) distribution in the epididymis and OXGR1-immunoreactive cells were identified as SMCs by double-labeling for OXGR1 and α-smooth muscle actin (α-SMA; red). Nuclei were stained with DAPI (blue). Scale bars = 200 μm. (d, e) Immunoblots (d) and quantification (e) of OXGR1 protein in the epididymis of 3-month-old and 12-month-old mice (*n* = 3 per group). (f, g) Immunoblots (f) and quantification (g) of OXGR1 protein in the epididymis of mice after 1, 2, and 3 h HS at 35°C (*n* = 5 per group). Results are presented as means ± SEM. *P < 0.05, **P < 0.01 and ***P < 0.001 by nonpaired Student’s *t*-test compared with control.[ AU: The captions for Figures 1, 3, 4, and 5 currently contains reference to color used within the figure. For accessibility purposes reference to color should be avoided. If possible please provide updated wording for the figure caption to remove reference to color.]

First, we confirmed the tissue distribution of OXGR1 by western blotting (WB) and found that OXGR1 is highly expressed in the epididymis, with moderate occurrence in the spleen, lung, kidney, and testis (Fig. 1a and b). Further, OXGR1 is expressed in different segments of the epididymis (caput, corpus, and cauda) and colocalized with α-smooth muscle actin (α-SMA), a symbolic marker for SMCs (Fig. 1c), but not with aquaporin 9 (AQP9), a symbolic marker for epididymal epithelial principle cells (Supplementary Fig. S1c).

Aging or heat stress impedes male fertility and reproduction [16]. We found that OXGR1 expression in the epididymis of 12-month-old mice was significantly decreased compared with that of 3-month-old mice (Fig. 1d and e). In addition, heat stress induced a time-dependent decline in OXGR1 expression in the epididymis. OXGR1 expression was lowest at 3 h after heat-stress induction and was 50% lower than its expression in the usual physiological condition (23°C ± 3°C) (Fig. 1f and g). These data suggest a vital role of OXGR1 in epididymal SMCs, which might be associated with reproduction dysfunction during aging or heat stress.

### OXGR1-GKO impairs epididymal lumen morphology and fertility

**Figure 2:**
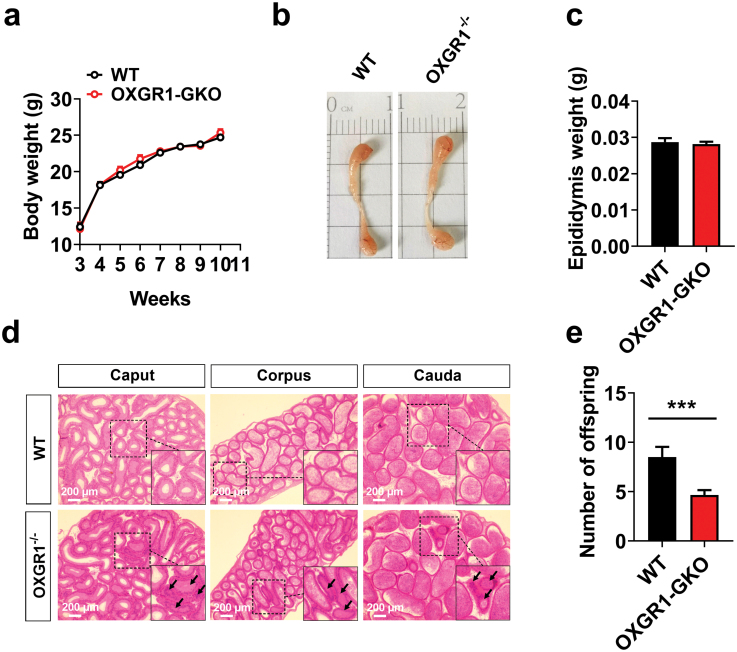
OXGR1-GKO damages epididymis morphology and male fertility. (a–c) Comparison of body weight (a), epididymal gross morphology (b), and epididymis weight (c) between male OXGR1-GKO and WT mice (*n* = 6). (d) Hematoxylin and eosin staining of representative epididymis sections from male OXGR1-GKO and WT mice. Original magnification: ×100. Scale bars = 200 μm. (e) Fertility test of male OXGR1-GKO and WT mice. Results are presented as means ± SEM (*n* = 6). ***P < 0.001 by nonpaired Student’s *t*-test compared with control.

To determine the role of OXGR1 in male reproduction, we applied the OXGR1-GKO strategy described in a previous study [12]. OXGR1 expression was validated by genotyping (Supplementary Fig. S1a), immunoblots (Supplementary Fig. S1b), and immunofluorescence analysis (Supplementary Fig. S1d). Although the body weight, the size, and the weight of the epididymis were unchanged (Fig. 2a−c), the morphology of the epididymal lumen in the OXGR1-GKO mice was distorted with a less epididymal lumen area (Fig. 2d). As expected, the fertility of the male OXGR1-GKO mice was considerably lower than that of the male wild-type (WT) mice (Fig. 2e).

### Effects of OXGR1-GKO on sperm morphology and function

**Figure 3:**
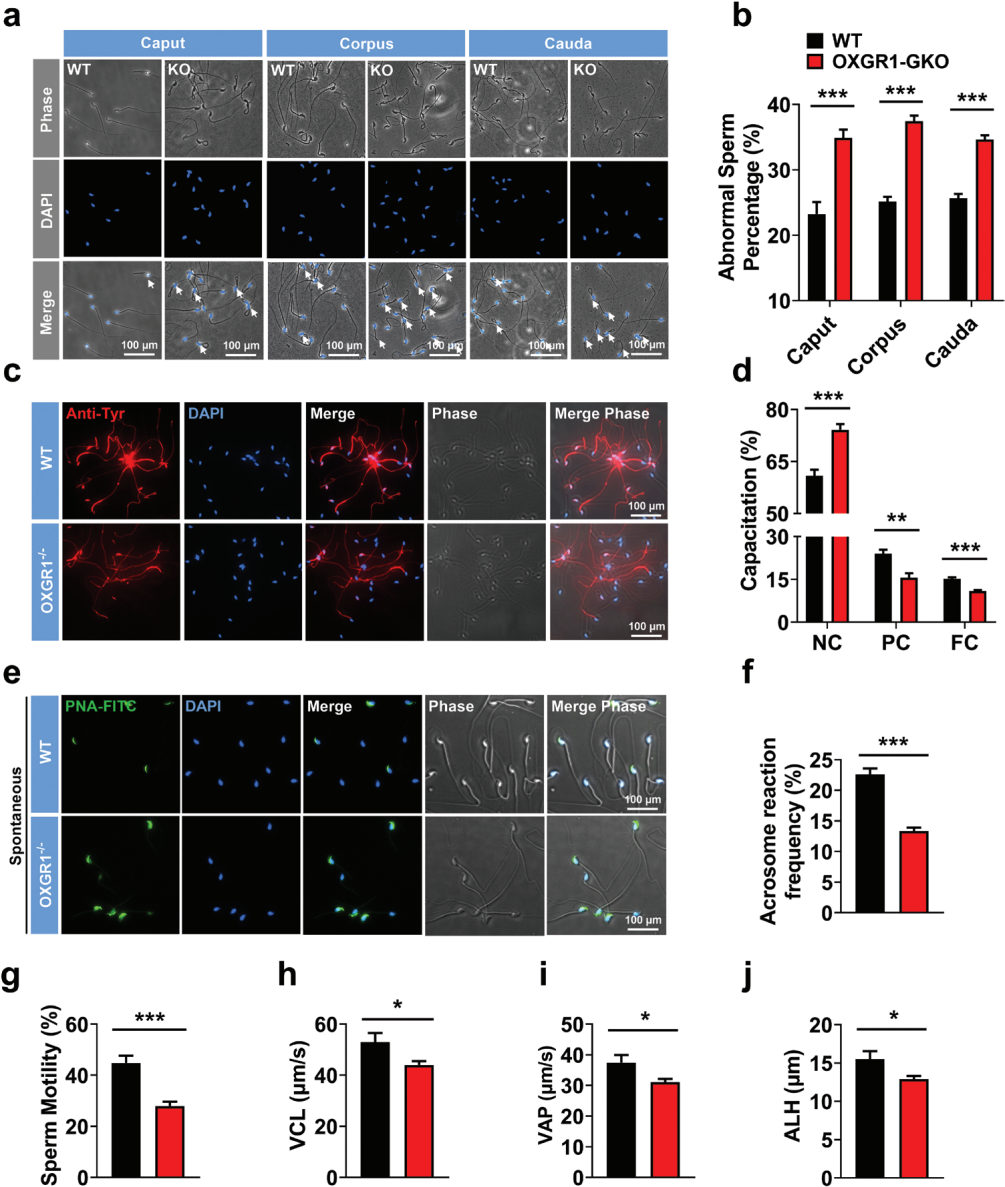
OXGR1-GKO impairs sperm morphology and sperm functions. (a) Phase-contrast and fluorescence staining images showing the impaired morphological defects in OXGR1-GKO sperm. The nucleus was stained with DAPI (blue). Scale bars = 100 μm. (b) Percentage of sperm with morphological defects in the caput, corpus and cauda epididymis from OXGR1-GKO mice. (c) Fluorescence staining images showing the total level of capacitation within individual sperm. Scale bars = 100 μm. (d) WT and OXGR1-GKO mice sperm tail tyrosine phosphorylation capacitation was measured at 1 h of *in vitro* capacitation. The sperm with three categories of capacitation were counted: non-capacitated (NC), partially capacitated (PC) and fully capacitated (FC). (e) Representative images of spontaneous acrosome reaction (AR) frequency in WT and OXGR1-GKO sperm. After sperm capacitation for 1 h, sperm were collected for PNA-FITC assay. Acrosome-positive sperm (green head) and acrosome-reacted sperm (no green in the head) in WT and OXGR1-GKO mice are shown under the specific conditions. Nuclei were stained with DAPI (blue). Scale bars = 100 μm. (f) Comparison of spontaneous acrosome reaction (AR) frequency between WT and OXGR1-GKO sperm. (g–j) Comparison of motility, curvilinear velocity (VCL), averaged path velocity (VAP) and amplitude of lateral head displacement (ALH) of sperm from WT and OXGR1-GKO mice. Data were shown as mean ± SEM (*n* = 6), *P < 0.05, **P<0.01 and ***P < 0.001 by nonpaired Student’s *t*-test compared with control.

Sperm gradually matures while passing through the epididymis [17]. This process endows sperm with the acquisition of motility and the potential for sperm capacitation and undergoing the acrosome reaction [2]. We further analyzed sperm morphology from the caput, corpus, and cauda segments of the epididymis. Compared with WT littermate mice, sperm from the caput to the cauda segments of the epididymis in OXGR1-GKO mice showed a dramatic increase in abnormal percentage with the typical morphological hairpin bending (Fig. 3a and b).

To clarify the potential sperm defects in OXGR1-GKO male mice, epididymal sperm were collected, incubated in capacitating medium for 1 h, and examined for the parameters of sperm motility at room temperature by performing computer-aided sperm analysis. The results revealed that OXGR1-GKO significantly reduced sperm motility (Fig. 3g), curvilinear velocity (VCL) (Fig. 3h), average path velocity (VAP) (Fig. 3i), and amplitude of lateral head displacement (Fig. 3j) without changing straight-line velocity (VSL) (Supplementary Fig. S2b) and beat cross frequency (Supplementary Fig. S2c). In addition, we collected cauda epididymis sperm from OXGR1-GKO mice and analyzed sperm capacitation according to previous studies [18, 19]. When OXGR1-GKO sperm were capacitated *in vitro*, the time-dependent increase in capacitation-associated protein phosphorylation was significantly decreased (Supplementary Fig. S2a). We further observed a significant decrease in the percentage of partial- or full-capacitated (PC or FC) sperm in the OXGR1-GKO group, whereas the percentage of non-capacitated (NC) sperm was significantly increased (Fig. 3c and d). The decreased capacitation-associated protein tyrosine phosphorylation also confirmed this observation in OXGR1-GKO mice (Supplementary Fig. S2a). Furthermore, to determine the capacity of OXGR1-GKO sperm acrosome reaction, sperm were stained with fluorescein isothiocyanate-conjugated peanut agglutinin (PNA-FITC) to investigate their capacity for the spontaneous acrosome reaction. Spontaneous acrosome reactions were significantly decreased in OXGR1-GKO mice compared with WT littermate mice (Fig. 3e and f).

### OXGR1 in the epididymis is required for normal sperm morphology and functions

**Figure 4:**
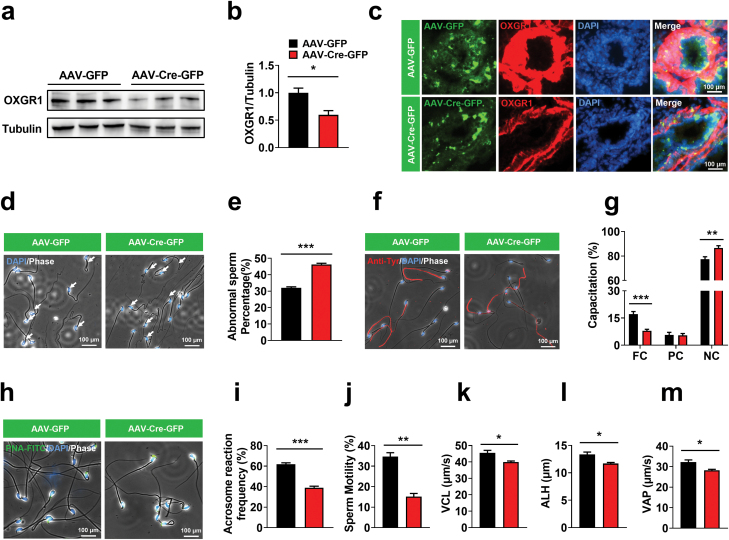
OXGR1 in the epididymis is required for normal sperm morphology and sperm functions. (a, b) The protein level of OXGR1 expression in the epididymis after injection of AAV-GFP and AAV-Cre-GFP (*n* = 3 per group). (c) Immunofluorescence showed the loss of OXGR1 in the epididymis after AAV-Cre-GFP injection. Scale bars = 100 μm. (d) Phase-contrast and fluorescence staining images showing the sperm morphological defects in cauda epididymis injected with AAV-GFP and AAV-Cre-GFP. The nucleus was stained with DAPI (blue). Scale bars = 100 μm. (e) Percentage of sperm with morphological defects in cauda epididymis after injection of AAV-GFP and AAV-Cre-GFP (*n* = 6 per group). (f) Fluorescence staining images showing the total level of capacitation within individual sperm. Scale bars = 100 μm. (g) Sperm tail tyrosine phosphorylation capacitation was measured at 1 h of *in vitro* capacitation after injection of AAV-GFP and AAV-Cre-GFP (*n* = 6 per group). (h) Representative images of spontaneous acrosome reaction (AR) frequency in cauda epididymis after injection of AAV-GFP and AAV-Cre-GFP. After sperm capacitation for 1 h, sperm were collected for PNA-FITC assay. Acrosome-positive sperm (green head) and acrosome-reacted sperm (no green in the head) in cauda epididymis after injection of AAV-GFP and AAV-Cre-GFP are shown under the indicated conditions. Nuclei were stained with DAPI (blue). Scale bars = 100 μm. (i) Comparison of spontaneous acrosome reaction (AR) frequency in cauda epididymis after injection of AAV-GFP and AAV-Cre-GFP (*n* = 6 per group). (j–m) Comparison of motility, VCL, VAP and ALH of sperm in the epididymis after injection of AAV-GFP and AAV-Cre-GFP (*n* = 3 per group). Data were shown as mean ± SEM, *P < 0.05, **P < 0.01 and ***P < 0.001 by nonpaired Student’s *t*-test compared with AAV-GFP injection.

To exclude the role of OXGR1 in other tissues, we further constructed an OXGR1-eKD model using OXGR1^Flox/Flox^ transgenic mice (Supplementary Fig. S3a and b) and AAV-Cre-GFP virus injection (Supplementary Fig. S3c). Both western blotting and immunofluorescence analysis confirmed the successful knockdown of OXGR1 in the epididymis (Fig. 4a−c), whereas OXGR1 protein expression in the testis was not affected (Supplementary Fig. S3d). Similar to those observed in OXGR1-GKO male mice, sperm tails in OXGR1-eKD mice presented hairpin bending at the annulus (Fig. 4d), and the percentage of abnormal sperm increased significantly compared with the AAV-GFP control (Fig. 4e). Subsequently, we collected sperm from the cauda epididymis injected with both adeno-associated viruses and incubated them in capacitation medium for 1 h to evaluate their capacitation. The results showed that the percentage of FC sperm in the epididymis injected with AAV-Cre-GFP decreased significantly, whereas the percentage of NC sperm increased remarkably compared with that injected with AAV-GFP (Fig. 4f and g), indicating that sperm capacitation was inhibited in OXGR1-eKD mice. Furthermore, the spontaneous acrosome reactions were significantly decreased in the epididymis injected with AAV-Cre-GFP compared with those injected with AAV-GFP (Fig. 4h and i). Sperm motility (Fig. 4j), VCL (Fig. 4k), VAP (Fig. 4l), and amplitude of lateral head displacement (Fig. 4m) were significantly decreased in the epididymis injected with AAV-Cre-GFP compared with those injected with AAV-GFP, whereas VSL (Supplementary Fig. S2d) and beat cross frequency (Supplementary Fig. S2e) did not change significantly. Interestingly, due to the influence of adeno-associated virus and the compensatory effect of OXGR1 expressed in other tissues other than the epididymis on sperm maturation, the effect of OXGR1-eKD on sperm maturation is different from that of OXGR1-GKO model. However, OXGR1-GKO and OXGR1-eKD data strongly support the conclusion that OXGR1 plays a pivotal role in epididymal sperm maturation.

### AKG effectively reverses sperm maturation disorder caused by aging and heat stress

**Figure 5:**
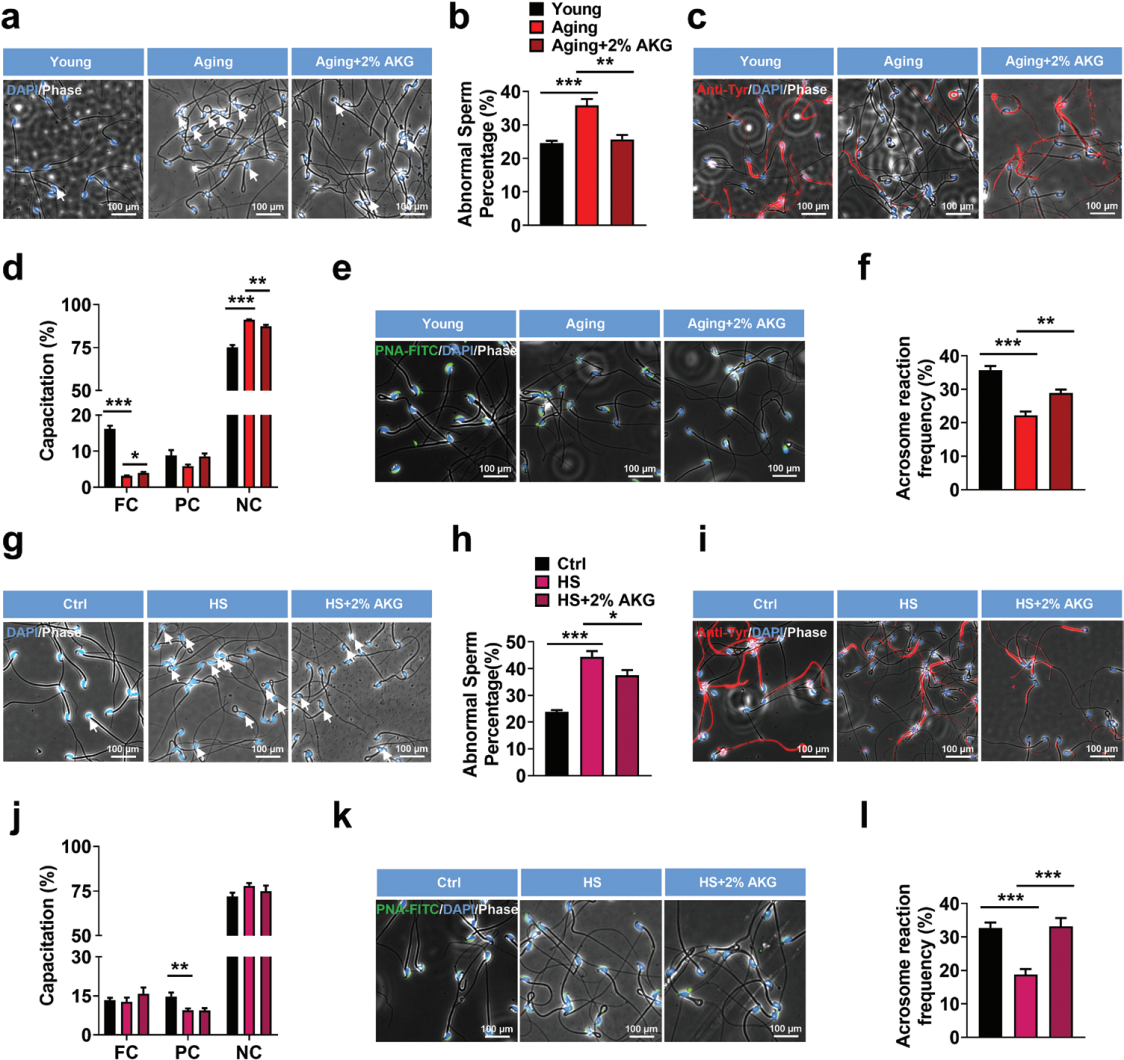
Drinking water supplemented with 2% AKG effectively reverses the sperm maturation disorder caused by aging and heat stress (HS). (a) Phase-contrast and fluorescence staining images showing the sperm morphological defects in cauda epididymis of young, aging and drinking water supplemented with 2% AKG (aging + 2% AKG) mice. The nucleus was stained with DAPI (blue). Scale bars = 100 μm. (b) Percentage of sperm with morphological defects in cauda epididymis of young, aging and aging + 2% AKG mice (*n* = 5 per group). (c) Fluorescence staining images showing the total level of capacitation within individual sperm in young, aging and aging + 2% AKG mice. Scale bars = 100 μm. (d) Young, aging, and aging + 2% AKG mice sperm tail tyrosine phosphorylation capacitation was measured at 1 h of *in vitro* capacitation (*n* = 5 per group). (e) Representative images of sperm spontaneous acrosome reaction (AR) frequency in young, aging and aging + 2% AKG mice. Scale bars = 100 μm. (f) Comparison of sperm spontaneous acrosome reaction (AR) frequency among young, aging and aging + 2% AKG mice (*n* = 5 per group). (g) Phase-contrast and fluorescence staining images showing the sperm morphological defects in cauda epididymis of WT (Ctrl), HS and HS drinking water supplemented with 2% AKG (HS + 2% AKG) mice. The nucleus was stained with DAPI (blue). Scale bars = 100 μm. (h) Percentage of sperm with morphological defects in cauda epididymis of Ctrl, HS and HS + 2% AKG mice (*n* = 6 per group). (i) Fluorescence staining images showing the total level of capacitation within individual sperm in Ctrl, HS and HS + 2% AKG mice. Scale bars = 100 μm. (j) Ctrl, HS and HS + 2% AKG mice sperm tail tyrosine phosphorylation capacitation was measured at 1 h of *in vitro* capacitation (*n* = 6 per group). (k) Representative images of sperm spontaneous acrosome reaction (AR) frequency in Ctrl, HS, and HS + 2% AKG mice. Scale bars = 100 μm. (l) Comparison of sperm spontaneous AR frequency among Ctrl, HS, and HS + 2% AKG mice (*n* = 6 per group). Data were shown as mean ± SEM, *P < 0.05, **P < 0.01 and ***P < 0.001 by two-way ANOVA followed by post hoc Bonferroni tests. In (b, d, f, h, j, and l).

As an endogenous agonist of OXGR1, AKG has broad therapeutic potential such as extending lifespan [20], maintaining intestinal health [21], decreasing the risk of obesity [12], and facilitating macrophage activation via epigenetic alteration [22, 23]. We observed a significant increase in abnormal sperm percentage and decreased sperm capacitation and spontaneous acrosome reaction frequency in aging mice (Fig. 5a-f). Drinking water supplemented with 2% AKG for 4 weeks effectively reduced abnormal sperm percentage (Fig. 5a and b) and increased sperm capacitation (Fig. 5c and d) and spontaneous acrosome reaction frequency (Fig. 5e and f) in aging mice.

For the heat stress model, mice exposed to heat stress at 60% relative humidity/35°C for 3 h significantly increased epididymal abnormal sperm percentage but decreased sperm capacitation and acrosome reaction frequency. However, drinking water supplemented with 2% AKG only reversed the effect of heat stress–induced increase in abnormal sperm percentage (Fig. 5g and h) and sperm acrosome reaction frequency reduction (Fig. 5k and l) but failed to recover the sperm’s partially capacitation reduction (Fig. 5i and j).

### Smooth muscle OXGR1 mediates the effect of AKG by regulating the acid–base balance of epididymal fluid

**Figure 6:**
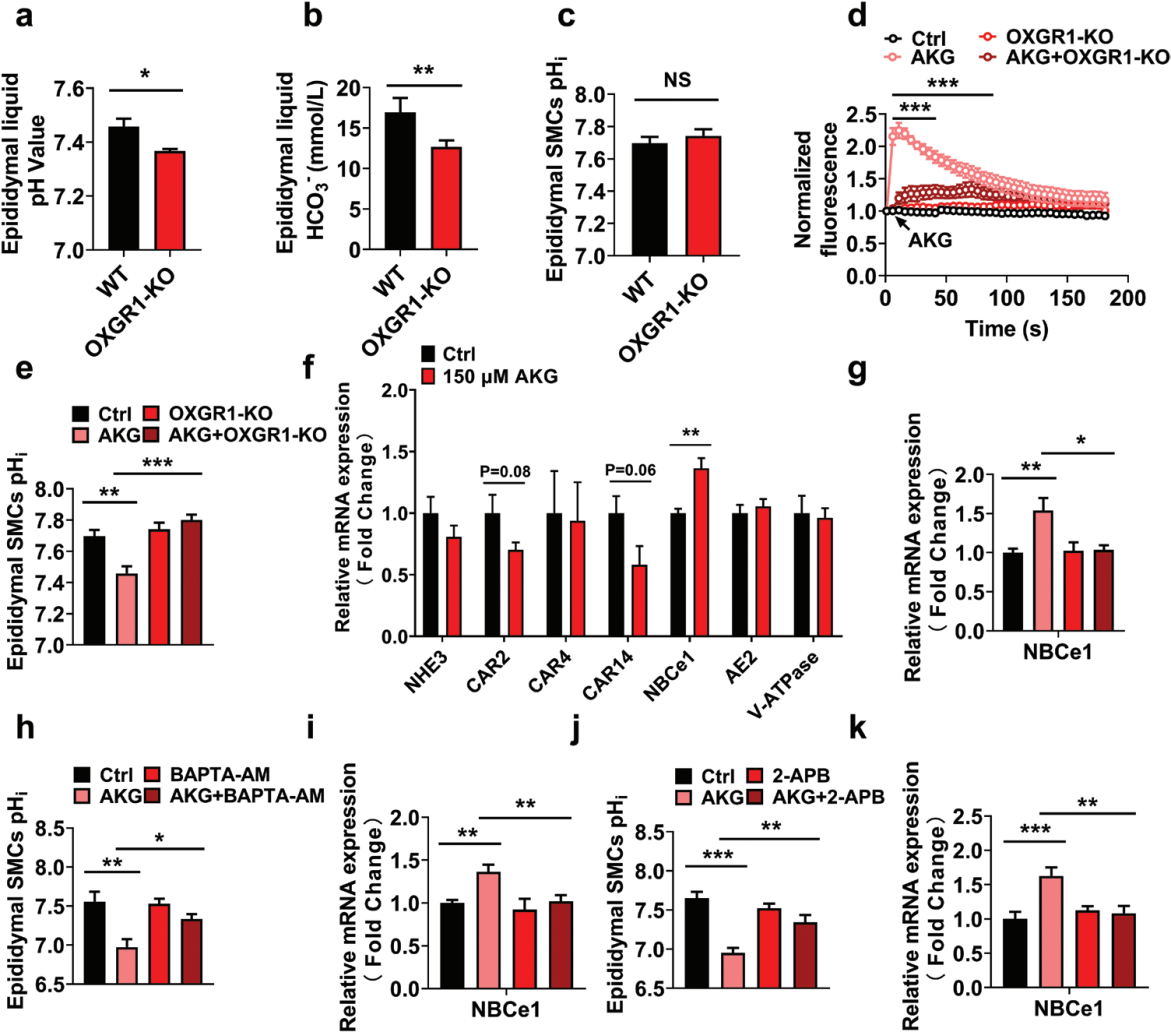
Smooth muscle OXGR1 mediates the effect of AKG by regulating the acid–base balance of epididymal fluid. (a, b) pH value and HCO_3_^−^ concentration in epididymal fluid of male WT and OXGR1-GKO mice. (a) pH value. WT: *n* = 4, OXGR1-GKO: *n* = 5. (b) HCO_3_^−^ concentration. WT: *n* = 4, OXGR1-GKO: *n* = 6. (c) pH values in epididymal SMCs of male WT and OXGR1-GKO mice (*n* = 8 per group). (d) Intracellular calcium ion [Ca^2+^] changes in epididymal SMCs of male WT and OXGR1-GKO mice cultured with HBSS buffer and 150 μM AKG (*n* = 6 per group). (e) pH values in epididymal SMCs of male WT and OXGR1-GKO mice cultured with HBSS buffer and 150 μM AKG (*n* = 8 per group). (f) The mRNA expression of acid–base transport-related genes in epididymal SMCs treated with HBSS buffer and 150 μM AKG. NHE3. Ctrl: *n* = 5, AKG: *n* = 6. CAR2. Ctrl: *n* = 8, AKG: *n* = 8. CAR4. Ctrl: *n* = 4, AKG: *n* = 4. CAR14. Ctrl: *n* = 8, AKG: *n* = 7. NBCe1. Ctrl: *n* = 7, AKG: *n* = 8. AE2. Ctrl: *n* = 7, AKG: *n* = 8. V-ATPase. Ctrl: *n* = 8, AKG: *n* = 6. (g) NBCe1 mRNA expression in epididymal SMCs of male WT and OXGR1-GKO mice cultured with HBSS buffer and 150 μM AKG. Ctrl: *n* = 10; AKG: *n* = 12; OXGR1-KO: *n* = 10; AKG + OXGR1-KO: *n* = 10. (h) pH values in epididymal SMCs cultured with HBSS + Ctrl, AKG (150 μM) + Ctrl, BAPTA-AM (50 μM) + Ctrl, BAPTA-AM + AKG (*n* = 7 per group). (i) NBCe1 mRNA expression in epididymal SMCs cultured with HBSS + Ctrl: *n* = 7, AKG (150 μM) + Ctrl: *n* = 8, BAPTA-AM (50 μM) + Ctrl: *n* = 6, BAPTA-AM + AKG: *n* = 8. (j) pH values in epididymal SMCs cultured with HBSS + Ctrl, AKG (150 μM) + Ctrl, 2-APB (5 μM) + Ctrl, 2-APB + AKG (*n* = 6 per group). (k) NBCe1 mRNA expression in epididymal SMCs cultured with HBSS + Ctrl, AKG (150 μM) + Ctrl, 2-APB (5 μM) + Ctrl, 2-APB + AKG (*n* = 12 per group). Data were shown as mean ± SEM, *P < 0.05 and **P < 0.01 by nonpaired Student’s *t*-test. In (a–c and f), ***P < 0.001 by nonpaired Student’s *t*-test. In (d), *P < 0.05, **P < 0.01 and ***P < 0.001 by two-way ANOVA followed by post hoc Bonferroni tests. In (e and g–k).

Epididymal SMCs play a crucial role in sperm maturation by promoting transepithelial K^+^ secretion to regulate the epididymal fluid microenvironment and ultimately affect sperm maturation [24]. To elucidate the potential mechanism underlying the action of OXGR1 in epididymal SMCs on the microenvironment of epididymal fluid, we used a blood gas analyzer to detect fluid parameters and revealed that OXGR1-GKO significantly reduced pH value and HCO_3_^−^ concentration of epididymal fluid (Fig. 6a and b), but exhibited no significant effect on the pH value in epididymal SMCs (Fig. 6c). Thus, OXGR1 may play a vital role in the transport of HCO_3_^−^ and H^+^.

Subsequently, we isolated the primary epididymal SMCs of WT and OXGR1-GKO mice (Supplementary Fig. S4a) and treated them with AKG. The results showed that AKG significantly promoted the release of instantaneous intracellular calcium in epididymal SMCs, whereas OXGR1-GKO could effectively abolish the stimulatory effects of AKG on instantaneous intracellular calcium in epididymal SMCs (Fig. 6d). Furthermore, we measured the intracellular pH (pH_i_) in epididymal SMCs using the fluorescent pH-sensitive dye BCECF-AM. The results demonstrated that AKG significantly reduced the pH_i_ in epididymal SMCs, whereas OXGR1-GKO effectively reversed a decrease in pH_i_ induced by AKG (Fig. 6e), suggesting that AKG may promote the release of instantaneous intracellular calcium in epididymal SMCs via the OXGR1 receptor, thereby changing the transport of H^+^ and HCO_3_^−^ and the acid–base environment of epididymal fluid.

**Figure 7:**
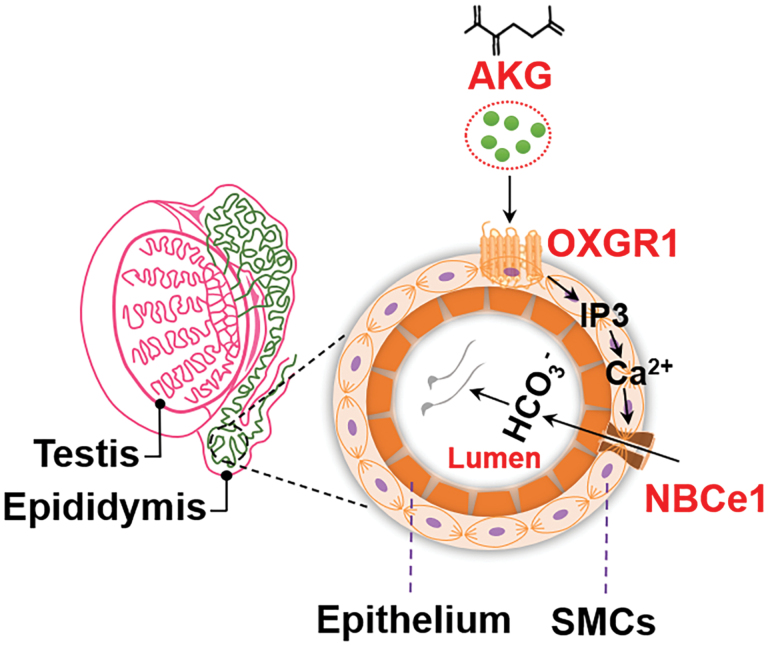
Schematic diagram of OXGR1-mediated AKG regulating the epididymal fluid acid–base balance. AKG upregulates the mRNA expression of the Na^+^–HCO_3_^−^ cotransporter NBCe1 by promoting the release of Ca^2+^ stored in the SR of epididymal SMCs by IP3 via OXGR1, thereby affecting HCO_3_^−^ secretion and epididymal fluid acid–base balance.

A previous study showed that Na^+^/H^+^ exchanger NHE3, Na^+^–HCO_3_^−^ cotransporter NBCe1, and Cl^−^/HCO_3_^−^ exchanger AE2 on the principal cells of the initial epididymal segment were involved in the transport of H^+^ and HCO_3_^−^ [25]. Therefore, we investigated whether OXGR1 mediates AKG to regulate the transport of H^+^ and HCO_3_^−^ through these ion transporters. Quantitative PCR (qPCR) results showed that AKG treatment significantly increased the mRNA expression of Na^+^–HCO_3_^−^ cotransporter NBCe1. However, AKG treatment did not significantly affect the mRNA expression of Na^+^/H^+^ exchanger NHE3 and Cl^−^/HCO_3_^−^ exchanger AE2 (Fig. 6f). To further investigate whether OXGR1 mediates AKG to increase the mRNA expression of NBCe1, primary epididymal SMCs were isolated and treated with AKG. We found that OXGR1-GKO effectively abolished the upregulation of mRNA expression of NBCe1 induced by AKG (Fig. 6g), suggesting that AKG/OXGR1-regulation of HCO_3_^−^ transport is dependent on the NBCe1 pathway. We further used the Ca^2+^ chelator BAPTA-AM to block the release of instantaneous Ca^2+^ in epididymal SMCs. Real-time qPCR analysis showed that instantaneous intracellular Ca^2+^ chelation could effectively abolish the effect of AKG on the mRNA expression of NBCe1 (Fig. 6i). AKG’s effect on pH reduction in epididymal SMCs disappeared when instantaneous intracellular Ca^2+^ signaling was blocked (Fig. 6h). Thus, intracellular Ca^2+^ signaling mediates the AKG/OXGR1 regulation of NBCe1 expression and changes in pH_i_. Since OXGR1 activates the β-isoforms of phospholipase C via Gq, and results in the release of sarcoplasmic reticulum (SR)-stored Ca^2+^ by inositol-1,4,5-trisphosphate (IP3) [26, 27], we used the IP3 receptor inhibitor 2-APB that regulates IP3-induced Ca^2+^ release to block Ca^2+^ release from the SR in epididymal SMCs, and found that 2-APB significantly reversed the effect of AKG-induced pH_i_ reduction (Fig. 6j) and NBCe1 mRNA upregulation (Fig. 6k). To conclude, AKG could promote the release of Ca^2+^ stored in the SR of epididymal SMCs by IP3 via OXGR1 and then upregulate the expression of Na^+^–HCO_3_^−^ transporter NBCe1, thereby affecting HCO_3_^−^ transport, and affecting epididymal fluid acid–base balance and sperm maturation (Fig. 7).

## Discussion

The main finding of this study is that OXGR1 is highly expressed in the mouse epididymis and is distributed in the SMCs of the mouse epididymis. Using OXGR1-GKO and OXGR1-eKD models, we showed that OXGR1 deficiency impaired epididymal sperm maturation and male fertility, including the abnormal epididymal structure and sperm morphology and reduced sperm functions and litter size. We observed that heat stress and aging could significantly decrease the expression of OXGR1 in the epididymis and thus damage epididymal sperm maturation, whereas AKG could prevent epididymal sperm maturation disorders caused by heat stress and aging. Furthermore, this study provides *in vitro* evidence supporting that AKG can upregulate the mRNA expression of the Na^+^–HCO_3_^−^ cotransporter NBCe1 by promoting the release of Ca^2+^ stored in the SR of epididymal SMCs by IP3 via OXGR1, thereby affecting HCO_3_^−^ secretion and epididymal fluid acid–base balance. These findings showed that epididymal OXGR1-positive cells are mainly SMCs that regulate sperm maturation by regulating the acid–base balance of the local epididymal microenvironment. Moreover, AKG, a key metabolic intermediate of the TCA, regulates epididymal sperm maturation via OXGR1 signaling.

OXGR1 is a G protein–coupled receptor located on the plasma membrane and participates in various physiological processes. Previous studies using qRT-PCR showed that human OXGR1 mRNA was mainly expressed in the trachea, salivary gland, kidney, and fetal brain, and was highly expressed in mast cells derived from umbilical cord blood [28]. We performed western blotting and immunofluorescence analysis to reveal that OXGR1 is mainly expressed in epididymal SMCs. SMCs are distributed in blood vessels, the digestive tract, and other tissues. OXGR1 may play a role in vascular responses in vascular SMCs distributed in the human nasal mucosa and may be involved in chronic signs and symptoms such as nasal polyps in patients with aspirin-exacerbated respiratory disease [28, 29]. Immunoreactive OXGR1 is located in the neurons of the myenteric plexus and nerve fibers running between SMCs [29], suggesting that OXGR1 may play an essential role in intestinal motivation and microbiota by activating the SMCs of the digestive tract. The epididymal canal is surrounded by a thin layer of SMCs, and previous studies have shown that OXGR1 is expressed only in narrow cells in the initial segment of the epididymis [9]; however, whether it is expressed in other cells of the epididymis and its specific functions require more attention.

OXGR1 is distributed in the epididymal SMCs, and OXGR1 global knockout is detrimental to epididymal sperm maturation. Since OXGR1 is expressed in tissues other than the epididymis, to confirm further the role of OXGR1 in epididymal SMCs, we developed the OXGR1-eKD model by injecting AAV-Cre-GFP into the epididymis of OXGR1^Flox/Flox^ mice. Interestingly, the effects of OXGR1-eKD and OXGR1-GKO on sperm maturation have both similarities and differences. On the one hand, it is further confirmed that epididymal OXGR1-positive cells are mainly SMCs that play a crucial role in sperm maturation. On the other hand, OXGR1 is a receptor expressed in multiple tissues. It is difficult to conclude the role of a multi-tissue expressed receptor by global knockout model, which depends on the expression site and endogenous ligand concentration. First, OXGR1 is highly expressed in adrenal medulla, which is involved in the secretion of serum epinephrine to reduce the demand for K^+^ in the later stage of sperm capacitation and acrosome reaction [30], thus mediating the beneficial effect on sperm maturation. Further, AKG is a key metabolite of the TCA cycle, which is also sensitive to local stimulus, such as viral infection. Therefore, these might be the reasons for the divergence between OXGR1-GKO and OXGR1-eKD models. Moreover, the difference between OXGR1-eKD and OXGR1-GKO models in basic level, such as abnormal sperm percentage, capacitation, spontaneous acrosome reaction frequency, and sperm motility, was mainly related to adeno-associated virus. Several publications have confirmed that the COVID-19 infection significantly increased the abnormal sperm percentage [31]; the Sendai virus attached to the sperm acrosome and induced a combination capacitation–acrosome reaction, which may improve sperm capacitation and acrosome reaction frequency [32]. Therefore, due to the presence of AAV-GFP virus, the basic level of sperm indicators was higher. Taken together, viral infection has a significant impact on male reproduction, which further highlights the potential role of AKG/OXGR1 system in the treatment of male reproductive decline caused by associated viral infection.

The well-regulated function of epididymal SMCs ensures the transit of spermatozoa through the epididymal duct, during which spermatozoa obtain motility and fertilizing capacity [33]. Previous studies have focused more on the relaxation and contraction of the epididymal smooth muscle. The relaxation of smooth muscle is usually mediated by cGMP signaling, and the components of this pathway are found within the male reproductive tract. In contrast, the contraction of cauda epididymal smooth muscle is governed by complex interactions among hormones, autacoids, and neurotransmitters released from the epididymal intramural nerve endings [33, 34]. α_1_-Adrenoceptors (α_1_-ARs) located in the cauda epididymal smooth muscle mediate the contractile effect of 5-hydroxytryptamine (5-HT) on epididymal smooth muscle, which plays an essential role during the seminal emission phase of ejaculation [33, 35]. Additionally, PDGFRα-positive interstitial cells (PDGFRα (+) ICs) are thought to be involved in controlling the movement of smooth muscle from the initial segment to the cauda epididymis via intercellular signaling [36]. The role of epididymal smooth muscle might be to facilitate sperm transport through contraction and relaxation while ignoring the other functions of epididymal smooth muscle critical to sperm maturation. Previous studies have indicated that OXGR1 is involved in the acid–base balance of renal tubular fluid [10]. The regulation of epididymal smooth muscle in the acid–base microenvironment of epididymal fluid may play an essential role in sperm maturation. Consistent with kidney tissue, OXGR1 knockout in epididymal smooth muscle resulted in a significant decrease in the pH value and HCO_3_^−^ concentration of epididymal fluid, and OXGR1 knockout could significantly reverse the effect of AKG-induced pH reduction in epididymal SMCs. These results suggest that OXGR1 may play a key role in the transport of H^+^ and HCO_3_^−^. HCO_3_^−^ in epididymal fluid plays a crucial role in the entire reproduction process in mammals, including spermatogenesis, sperm capacitation, fertilization, and development of early-stage embryos [37]. Therefore, it is further confirmed that the epididymal smooth muscle can regulate the sperm maturation by regulating the acid–base balance of epididymal fluid. OXGR1 expressed in type B (and in non-A-non-B-type) ICs of the distal nephron and collecting tube could mediate the AKG regulation of HCO_3_^−^ secretion and electroneutral transepithelial NaCl reabsorption [10]. However, it is not clear whether the AKG/OXGR1 system regulates the acid–base balance of renal tubular fluid via the acid–base transporter. Previous studies have reported that the principal cells of the proximal regions of the epididymis are fully equipped for HCO_3_^−^ reabsorption, and they use transport mechanisms that are very similar to those used by the proximal kidney tubule. In the initial segments of the epididymis, the apical Na^+^/H^+^ exchanger NHE3 secretes protons into the epididymal lumen, whereas the Na^+^–HCO_3_^−^ cotransporter NBCe1 or anion exchanger AE2 participates in the reabsorption of HCO_3_^−^ [25]. Therefore, we hypothesized that the AKG/OXGR1 system might regulate the acid–base balance of epididymal fluid by affecting the expression of these acid–base transporters. As expected, we found that AKG could increase the mRNA expression of Na^+^–HCO_3_^−^ cotransporter NBCe1 via OXGR1 in epididymal SMCs, and this effect was mainly mediated by SR calcium signaling. Previous studies have found that cholinergic agonists significantly increased intracellular Ca^2+^ [38] and stimulated protein kinase C (PKC)-dependent NBCe1 expression [39]. Therefore, we speculated that the AKG/OXGR1 system may upregulate NBCe1 expression by stimulating Ca^2+^–PKC pathway, and ultimately affect the acid–base balance of epididymal fluid. NBCe1-defective mice causes 100% mortality before day 25 [40], which suggests that smooth muscle AKG/OXGR1-NBCe1 pathway may play a momentous role in the therapy of male infertility. Epididymal SMCs regulate sperm maturation by regulating the acid–base balance of epididymal fluid, rather than by contraction and relaxation. This provides a valuable reference for exploring the role of smooth muscle in epididymal sperm maturation.

Most studies have reported that aging reduces testosterone secretion and spermatogenesis in male animals and adversely affects sperm motility acquisition, sperm morphology, and sperm quality [41]. In addition, heat stress damages spermatogenic cells of male animals through apoptosis, autophagy, DNA damage, and excessive production of reactive oxygen species [42]. However, a few studies have shown the molecular mechanism and regulation of sperm dysfunction caused by aging and heat stress. In this study, we found that aging and heat stress significantly reduced the expression of epididymal OXGR1 and impaired sperm maturation, whereas drinking water supplemented with 2% AKG effectively reversed sperm maturation disorder caused by aging and heat stress. Moreover, previous studies have reported that AKG reduces the average physiological age by 8 years [43], thus promoting a longer and healthier life span [44]; however, AKG’s resistance to aging remains unclear. Theoretically, the decreased fecundity and an increased risk for disturbed pregnancies occur with advancing paternal age [5], and it is found that aging significantly reduced serum AKG levels [45]. Therefore, our study provides an idea that AKG may play the role of “aging switch” via OXGR1 in SMCs, and the smooth muscle AKG/OXGR1 signaling has a potential therapeutic effect on the decrease in male fertility caused by aging. Moreover, epididymal injection of adeno-associated virus, even AAV-GFP control virus, adversely affected the functions of epididymal sperm, which is consistent with the clinically observed decrease in male fertility caused by viral infection. Our study provides evidence that smooth muscle AKG/OXGR1 signaling may have a potential value in treating male infertility caused by related viral infection. Finally, this study has theoretical significance in revealing the molecular mechanism of exercise metabolic intermediates regulating sperm maturation and provides a reference for the development of critical functional nutrients regulating male reproduction.

## Materials and methods

### Animal experiments

C57BL/6J male mice were purchased from the Animal Experiment Center of Guangdong Province (Guangzhou, Guangdong, China). OXGR1-GKO mice and OXGR1^Flox/Flox^ mice were obtained from Shanghai Research Center for Model Organisms (Shanghai, China) and Beijing Viewsolid Biotech Co. Ltd (Beijing, China), respectively. They were generated and maintained on a C57BL/6J background. The OXGR1^Flox/Flox^ male mice were injected AAV-Cre-GFP at 8 weeks to induce the deleting of OXGR1 in the epididymis. Mice were housed in a temperature controlled at 23°C ± 3°C and humidity controlled at 70% ± 10% for a 12-h light/12-h dark cycle. Unless otherwise noted, the mice had *ad libitum* access to standard mouse chow and water. All experimental male mice were fed in accordance with “The Guidelines on Nursing Experimental Animals” issued by the Ministry of Science and Technology, PRC, and approved by the College of Animal Science, South China Agricultural University.

For chronic AKG-treated experiment, 10 12-month-old male mice were randomly divided into two groups according to the principle of consistent body weight (5 mice per group). They were singly housed and randomly assigned to receive water or water supplemented with 2% AKG (α-ketoglutaric acid disodium salt, A610289, Sangon Biotech [Guangzhou] Co., Ltd). Body weight and food intake were monitored weekly for 4 weeks. Epididymal sperm of aging male mice were collected for sperm morphological analysis, sperm capacitation analysis, and sperm acrosome reaction analysis. In addition, epididymis of 12-month-old male mice was collected, and OXGR1 expression was measured by western blot analysis and immunofluorescence staining.

For another chronic AKG-treated experiment, 12 10-week-old male mice were randomly divided into two groups according to the principle of consistent body weight (6 mice per group). One day before the heat stress test, the mice were singly housed and randomly assigned to receive water or water supplemented with 2% AKG. At the beginning of the experiment, the mice were placed in an intelligent artificial climate chamber (HWS-317, Ningbo Dongnan Instrument Co., Ltd, China) which was temperature controlled at 35°C and humidity controlled at 60% for 3 h to undergo acute heat stress. Epididymal sperm were collected for further analysis after a heat stress test. Additionally, under the temperature controlled of 35°C , the effects of different heat stress duration on OXGR1 expression were detected by WB and immunofluorescence.

### OXGR1 global knockout mouse model

OXGR1-GKO mouse model was generated by Shanghai Model Organisms Center, Inc. The OXGR1-GKO mouse model was established as described before [12]. Briefly, OXGR1-GKO mouse model was established using the clustered regularly interspaced short palindromic repeats (CRISPR) approach. The mRNA expression of OXGR1 was compared between WT and OXGR1-GKO mice by PCR (Fig. S1A). Homozygous OXGR1-GKO mice were obtained by hybridization of F1 mice with exon 4 protein-coding region deletion.

### Gene types identify and generation of mice with ablation of OXGR1 in the epididymis

The obtained mouse tails were genotyped by PCR and gel electrophoresis. For verification of the OXGR1^Flox/Flox^ alleles, the PCR products were amplified using the primers 5ʹ-GCCAGAGGATTCAGAATGGTATC-3ʹ and 5ʹ-TCTAACTTCCACAGGCACACT-3ʹ (KI-floxp: 197 bp, WT: 163 bp); 5ʹ-CTTAAAGGCTCGAAGGCTA-3ʹ and 5ʹ-GTGAATGATGCGTGGCTGTT-3ʹ (KI-floxp: 205 bp, WT: 171 bp).

To generate epididymal-specific OXGR1 knockdown mice, 8-week-old OXGR1^Flox/Flox^ mice were injected with AAV2/9-CMV_bGI-Cre-EGFP-pA (Titer: 10^13^ v.g/ml, Taitool Bioscience Co. Ltd, Shanghai, China) to induce the deleting of OXGR1 in epididymal SMCs. In addition, AAV2/9-CMV-GFP (Titer: 10^12^ v.g/ml, Hanbio Biotechnology Co., Ltd. Shanghai, China) was injected into the opposite epididymis of the same OXGR1^Flox/Flox^ mouse as a negative control. Two weeks after injection, the mice were sacrificed and sperm samples were collected for further analysis.

### Isolation and culture of primary epididymal SMCs

Five-week-old WT/OXGR1-GKO mice were anesthetized with isoflurane and sacrificed. All remaining procedures were performed in a biosafety cabinet with aseptic techniques. After the removal of connective tissue around the epididymis, the epididymis was transferred to DMFM/F12 medium (Gibco, USA) containing 20% FBS (Gibco, USA) and cut into small pieces. The tissue fragments were transferred to cell dissociation solution (7.5 mg collagenase type II was dissolved in 5 ml DMFM/F12 medium containing 20% FBS) and shaken in a 37°C constant temperature shaking shaker (H25-H, Jintan Ronghua Instrument Manufacturing Co., Ltd, China) for 2 h. Then, the digestive juice was filtered through a 100-μm cell strainer (Millipore, USA) and centrifuged at 800 *g* for 5 min. After centrifugation, the cells were seeded in 6-well cell plates (Corning, USA) and cultured in DMFM/F12 medium containing 20% FBS, 100 U/l penicillin (Sigma, USA), and 100 μg/ml streptomycin (Sigma, USA) at 37°C in a humidified incubator containing 5% CO_2_. Collagenase type II was purchased from Gibco.

### Western blot assay

Western blot assay was performed as described previously [46]. Briefly, total protein was extracted from epididymis samples using RIPA lysis buffer that contained 1 mM phenylmethylsulfonyl fluoride (PMSF). Total protein concentration was determined using a BCA protein assay kit (Thermo Fisher Scientific). After separation on 10% sodium dodecyl sulfate-polyacrylamide gel electrophoresis (SDS-PAGE) gels, the proteins were transferred to polyvinylidene fluoride (PVDF, Millipore, USA) membranes and then blocked with Everyblot Blocking Buffer (12010020, Bio-Rad, USA) for 5 min at room temperature. Subsequently, the PVDF membranes were probed with the indicated primary antibodies, including rabbit anti-OXGR1 (1:1000, ab140630, Abcam, UK), mouse anti-Tyrosine phosphorylation (1:10,000, clone 4G10, Millipore, USA), rabbit anti-α-Tubulin. Primary antibodies incubation was performed at 4°C overnight and followed by incubations with the goat anti-rabbit or mouse horseradish peroxidase (HRP)-conjugated secondary antibody (1:50,000, BS13278 or BS12478, Bioworld, USA) for 1 h at room temperature. Protein expression was measured with a Fluorescence Imaging System (ProteinSimple, Santa Clara, CA, USA) and normalized to α-Tubulin expression.

### Immunofluorescence staining

For immunofluorescence costaining, epididymal sections were incubated with the primary rabbit anti-OXGR1 antibody (1:1000, ab140630, Abcam, UK), mouse anti-α-SMA antibody (1:1000, sc-56499, Santa Cruz, USA) and anti-AQP9 antibody (1:50, 862723, ZenBio, China) at room temperature overnight, followed by FITC-conjugated goat anti-rabbit/mouse (1:500, 111-095-003/115-095-003, Jackson ImmunoResearch, USA) or Cy3-conjugated goat anti-mouse/rabbit (1:500, 115-165-003/111-165-003, Jackson ImmunoResearch, USA) secondary antibodies at room temperature for 1 h. Sections were mounted on slides and coverslipped with Mounting Medium with DAPI (0100-20, SouthernBiotech, USA). Fluorescent images were taken using a Nikon Eclipse Ti-S microscope (Nikon Instruments, Tokyo, Japan).

### RNA extraction and quantitative RT-PCR (qRT-PCR)

Epididymal SMCs were pretreated with 50 μM Ca^2+^ chelator BAPTA-AM (A1076, Sigma, USA) or 5 μM IP3 receptor inhibitor 2-APB (S6657, Selleck, USA) for 1 h prior to AKG treatment. Total RNAs were extracted from basal, 150 μM AKG-treated (treatment for 24 h), BAPTA-AM/150 μM AKG-treated, or OXGR1-GKO/150 μM AKG-treated epididymal SMCs using the Hipure Universal RNA Mini Kit (R4130-02, Magen, China) according to the manufacturer’s instructions. Total RNA (2 µg) was reversed to cDNA with random primers using the M-MLV enzyme (Promega, USA). SYBR Green master mix reagents (Q711, Vazyme, China) and both sense/antisense primers were used for real-time qRT-PCR. qRT-PCR reaction was performed in QuantStudio 3 Flex Real-Time PCR system (7300HT, Applied Biosystems, USA). Normalized mRNA expression was calculated using the 2^-ΔCt^method, and *β*-actin was used as a candidate housekeeping gene. The list of primer sequences is presented in Supplementary Table S1.

### Hematoxylin and Eosin (HE) staining

HE staining was performed as described before [47]. Briefly, epididymis was fixed with animal testicular tissue fixative fluid (G1121, Servicebio, China) for 24 h and then dehydrated with graded ethanol, vitrified by dimethylbenzene, and embedded in the paraffin. Then the paraffin embedding epididymis was cut into sections with a thickness of 5 μM and stained with HE. Pictures of stained epididymis tissue were obtained in the same location.

### Fertility assay

Fertility test was performed as described previously [48]. Briefly, male mice were mated with 8-week-old female mice at a 1:2 ratio, in which one male and two females spent the night in a cage, and vaginal smears of the females were taken the next morning and examined under a microscope. Sperm smear-positive female mice were considered to have mated successfully, and these mice were then housed individually. The number of offspring per litter was recorded after the pups were born.

### Sperm morphology analysis

Sperm morphology analysis was performed as described previously [48]. Briefly, sperm were obtained from the caput, corpus, and cauda epididymis of WT, OXGR1-GKO, OXGR1-eKD, aging, and heat-stressed mice. Then, sperm were dispersed in a TYH medium (M2050, Nanjing Aibei Biotechnology Co., Ltd, China). Sperm were spread on slides and fixed in 4% paraformaldehyde (PFA) for 30 min. Finally, sections were mounted on slides and coverslipped with Mounting Medium with DAPI. Phase and fluorescent images were obtained using a Nikon Eclipse Ti-S microscope (Nikon Instruments, Tokyo, Japan).

### Sperm capacitation assay

Sperm capacitation assay was performed as described before [19]. Briefly, to assess the rate of capacitation-associated tyrosine phosphorylation, sperm were collected from cauda epididymis of WT and OXGR1-GKO mice and incubated in TYH medium (M2050, Nanjing Aibei Biotechnology Co., Ltd, China). Sperm were incubated in a TYH medium for 0, 0.5, and 1 h at 37°C in a humidified atmosphere containing 5% CO_2_. Following incubation, sperm were centrifuged and washed once with PBS. Then, total protein was collected from these sperm and used for western blot assay.

To assess the total level of capacitation within individual sperm, sperm were incubated for 0 or 1 h as described above. Sperm were spread on slides and fixed in 4% PFA for 30 min, then permeabilized in 0.2% Triton X-100 (Sigma, USA)/PBS for 1 h at room temperature. Sperm were incubated with the primary mouse anti-Tyrosine phosphorylation (1:1000, clone 4G10, Millipore, USA) at room temperature overnight, followed by Cy3-conjugated goat anti-mouse (1:500, 115-165-003, Jackson ImmunoResearch, USA) secondary antibodies at room temperature for 1 h. Sections were mounted on slides and coverslipped with Mounting Medium with DAPI. Phase and fluorescent images were taken using a Nikon Eclipse Ti-S microscope (Nikon Instruments, Tokyo, Japan), wherein at least 200 sperm cells per mouse were scored for capacitation based on sperm tail staining status: capacitated: full length of tail labeled; partially capacitated: part of sperm tail labeled; NC: no labeling of the tail.

### Sperm acrosome reaction analysis

Sperm acrosome reaction was evaluated as described previously [49]. Briefly, sperm were capacitated at 37°C in a humidified atmosphere containing 5% CO_2_. Then, sperm were pelleted and spread on slides to dry and fixed with 4% PFA. The acrosome was stained using 25 μg/ml FITC-conjugated peanut agglutinin (PNA-FITC, L7381, Sigma, USA) and placed in a dark humidified chamber at 4°C for 30 min. Finally, sections were mounted on slides and coverslipped with Mounting Medium with DAPI, wherein at least 200 sperm cells per mouse were randomly scored. Sperm cells with complete acrosome staining were identified as sperm without acrosome reaction. In contrast, sperm cells without acrosome staining were identified as sperm for acrosome reaction.

### Sperm motility analysis

Sperm motility was assayed as described before [2]. Briefly, sperm were collected from the cauda epididymis of WT/OXGR1-GKO mice and incubated *in vitro* for 1 h. Then, sperm motility was assessed using a computer-assisted sperm analyzer (CASA). All movement parameters, including sperm motility, VSL, VCL, and VAP were measured by CASA (ML500JZ, Nanning Songjing Tianlun Biotechnology Co., Ltd, China).

### Blood gas analysis

The collected 5 µl cauda epididymal fluid from WT/OXGR1-GKO mice was dissolved and diluted in 195 µl diluent, which was heated with H_3_PO_4_ and then adjusted to neutral pH with Ca (OH)_2_ to minimize alkali storage in the diluent. To reduce the impedance of the solution, 1mM NaHCO_3_ was added to increase the conductivity of the solution. Then, a blood gas analyzer (RAPIDPOINT 500, Siemens, USA) was used to measure pH, HCO_3_^−^ and other ion concentrations in the solution.

### Measurement of intracellular pH in epididymal SMCs

Intracellular pH in epididymal SMCs was measured as described before [50]. Briefly, the pH_i_ measurements were assessed with BCECF-AM. Epididymal SMCs were digested and adjusted to 1 × 10^6^ cells/ml with DMFM/F12 medium. 5 µM BCECF-AM (216254, Sigma, USA) was added and incubated at 37°C in a humidified atmosphere containing 5% CO_2_ for 1 h. Afterwards, the cells were pelleted and washed twice to remove free dye. To measure the pH_i_, the fluorescence signal was detected by a luminescence spectrometer (BioTek, USA). The fluorometric data were measured at excitation and emission wavelengths of 490/440 nm and 535 nm, respectively. Calibration was performed as described before [51].

### Measurement of intracellular Ca^2+^ in epididymal SMCs

Intracellular Ca^2+^ in epididymal SMCs was tested as described previously [12]. Briefly, epididymal SMCs were seeded into 24-well plates at a density of 1 × 10^5^ cells/cm^2^. When the cell fusion reached 50%, cells were washed twice with HBSS buffer and incubated with 10 μM Fluo-8-AM (21096, AAT Bioquest, USA) at 37°C in a humidified atmosphere containing 5% CO_2_ for 1 h. Then, cells were washed twice again with HBSS buffer and incubated with 150 μM AKG. Nikon Eclipse Ti-S microscope was used to observe fluorescence which was initiated by AKG. Fluorometric data were obtained at excitation and emission wavelengths of 490 and 525 nm respectively every 5 s over a 180-s period.

### Statistics

Statistical analyses were performed using GraphPad Prism 8.0 (GraphPad Software, San Diego, California, USA). Methods of statistical analyses were chosen based on the design of each experiment and are indicated in the figure legends. The data are presented as the means ±SEM. *P* ＜0.05 was considered to be statistically significant.

## Supplementary Material

loac012_suppl_Supplementary_Material
