# Supplementary material for: Smooth muscle AKG/OXGR1 signaling regulates epididymal fluid acid–base balance and sperm maturation
Source: Life Metab. 2022 Jul 14;1(1):67–80. doi: 10.1093/lifemeta/loac012 (PMC11749693; doi:10.1093/lifemeta/loac012)
Supplement: loac012_suppl_Supplementary_Material [file loac012_suppl_Supplementary_Material.pdf]

Smooth Muscle AKG/OXGR1 Signaling Regulates Epididymal Fluid Acid-base Balance and Sperm Maturation

Chang Xu[1, †], Yexian Yuan[1], Cha Zhang[1], Yuchuan Zhou[2], Jinping Yang[1], Huadong Yi[3], Ishwari Gyawali[1], Jingyi Lu[1], Sile Guo[1], Yunru Ji[1], Chengquan Tan[1], Songbo Wang[1], Yongliang Zhang[1], Qingyan Jiang[1, *], and Gang Shu[1, *]

[1]Guangdong Laboratory for Lingnan Modern Agriculture, National Engineering Research Center for Breeding Swine Industry and Guangdong Province Key Laboratory of Animal Nutritional Regulation, College of Animal Science, South China Agricultural University, Guangzhou, Guangdong 510642, China

[2]International Peace Maternity and Child Health Hospital, Shanghai Key Laboratory of Embryo Original Diseases, School of Medicine, Shanghai Jiao Tong University, Shanghai 200030, China

[3]State Key Laboratory of Biocontrol, Guangdong Provincial Key Laboratory for Aquatic Economic Animals, School of Life Sciences, Sun Yat-Sen University, Guangzhou 510275, China

†Lead author

*Correspondence to: Gang Shu, E-mail: shugang@scau.edu.cn; Qingyan Jiang, E-mail: qyjiang@scau.edu.cn

Supplementary Figure. S1. The validation of OXGR1-GKO and epididymal OXGR1 colocalisation staining.

(a) OXGR1-GKO allele was identified using PCR-based genotyping. (b) Immunoblots of OXGR1 protein in the epididymis of WT and OXGR1-GKO mice. (c) Co-localisation of OXGR1 (red), α-SMA (green) and AQP9 (red) in the epididymis. SMCs were stained with α-SMA (green), epididymal epithelial principle cells were stained with AQP9 (red). L: lumen, SM: smooth muscle, PC: principle cells, In: interduct compartment. Scale bars = 100 μm. (d) Deletion of the OXGR1 gene results in loss of all green fluorescence. SMCs were stained with α-SMA (red). Nuclei were stained with DAPI (blue). Scale bars = 100 μm.

Supplementary Figure. S2. Detection of sperm capacitation-associated protein tyrosine phosphorylation and sperm motility.

(a) Measurement of capacitation-associated protein tyrosine phosphorylation of sperm from WT and OXGR1-GKO mice. Sperm were incubated in capacitating medium and collected for Western blot per 0.5 h. α-Tubulin served as the loading control. (b, c) Comparison of straight-line velocity (VSL) and beat cross frequency (BCF) of sperm from WT and OXGR1-GKO mice (n = 6 per group). (d, e) Comparison of straight-line velocity (VSL) and beat cross frequency (BCF) of sperm in the epididymis after injection of AAV-GFP and AAV-Cre-GFP (n = 3 per group). Data were shown as mean ± SEM.

Supplementary Figure. S3. Principle and genotyping of Floxp knock-in and schematic experimental strategy of epididymal-specific OXGR1 knockdown.

(a) Schematic experimental strategy of Clustered Regularly Interspaced Short Palindromic Repeats (CRISPR) with sgRNAs targeting OXGR1 gene. According to OXGR1 genome structure and functional conserved regions of protein, FloxP sites will be inserted on both sides of Exon4 to achieve conditional knockout of Exon4. Conditional deletion of Exon4 will destroy functional conserved region 7tmA_OXGR1 and inactivates the protein. (b) OXGR1$^{Flox/Flox}$ allele was identified using PCR-based genotyping. Homozygous (HOM): 197/205 bp; WT: 163/171 bp; Heterozygote (HET): 197/205 bp and 163/171 bp. (c) Schematic experimental strategy to generate epididymal-specific OXGR1 knockdown mice. AAV-GFP and AAV-Cre-GFP viruses were simultaneously injected into the epididymis of 8-week-old OXGR1$^{Flox/Flox}$ homozygous mice, and epididymal-specific OXGR1 deletion mouse model was generated after 14 days of virus infection. (d) The protein level of OXGR1 expression in testis after epididymis injection of AAV-GFP and AAV-Cre-GFP.

Supplementary Figure. S4. Localisation of OXGR1 in epididymal SMCs and OXGR1-GKO cells validation.

(a) Immunofluorescence detection of OXGR1 (red) distribution in epididymal SMCs and most OXGR1-immunoreactive cells were identified as SMCs by double-labeling for OXGR1 and α-SMA (green). Nuclei were stained with DAPI (blue). Scale bars = 200 μm.

Supplementary Figure. S1

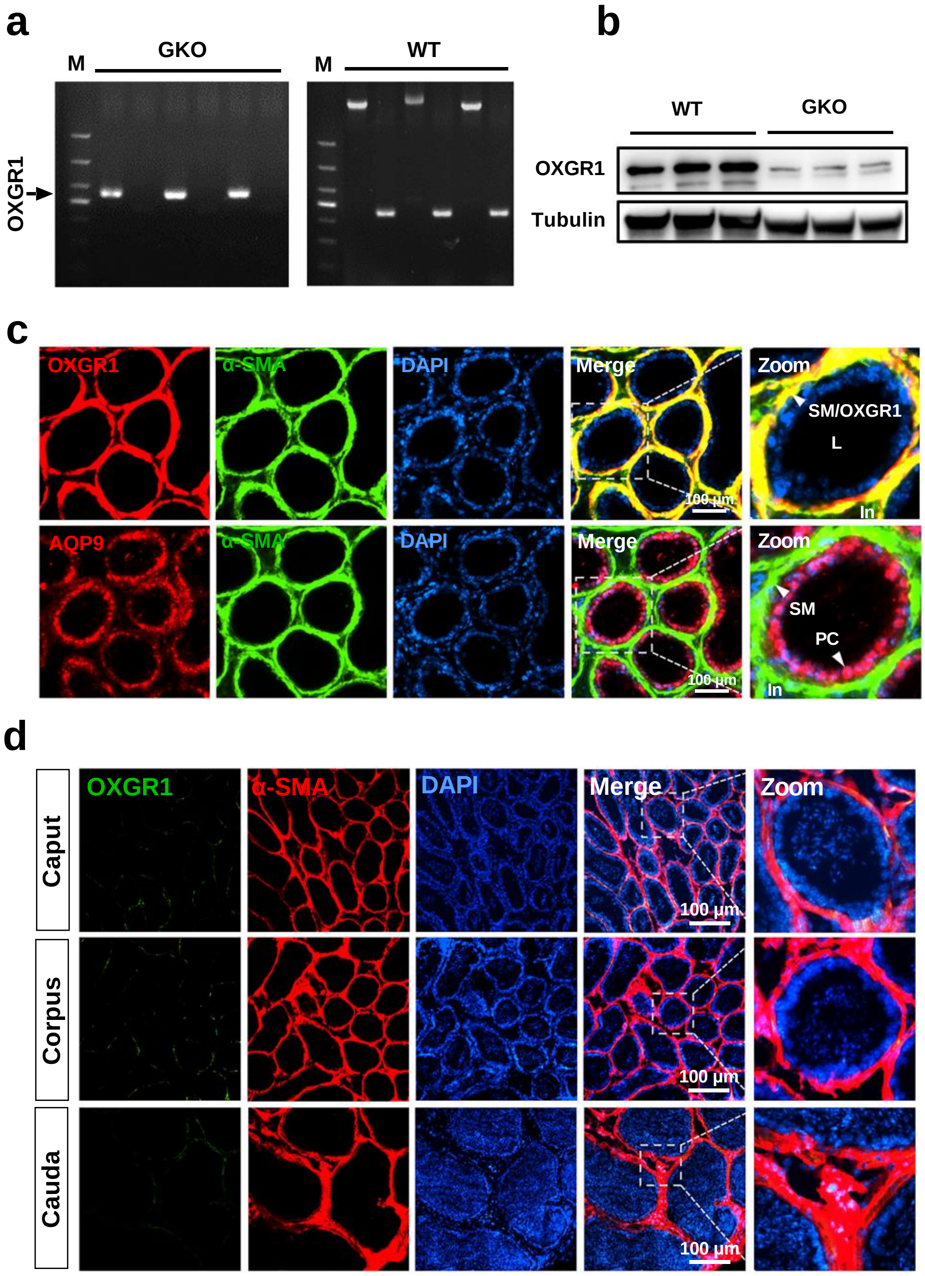

Supplementary Figure. S2

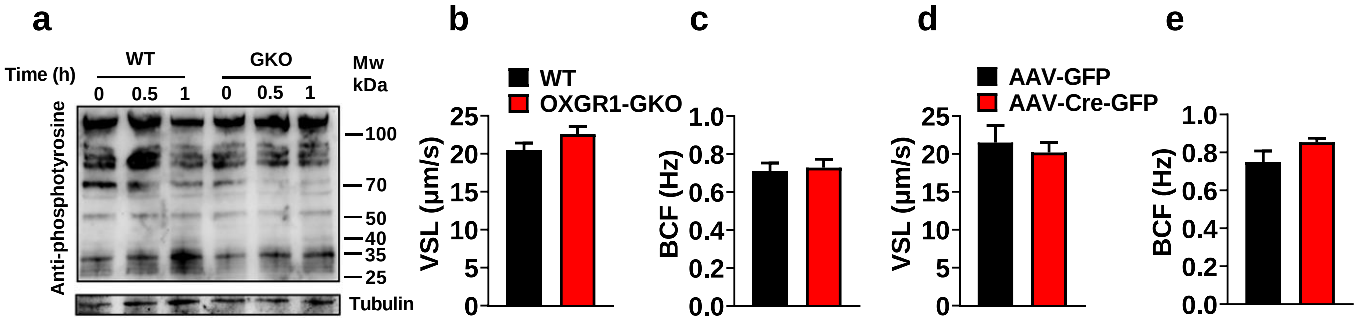

Supplementary Figure. S3

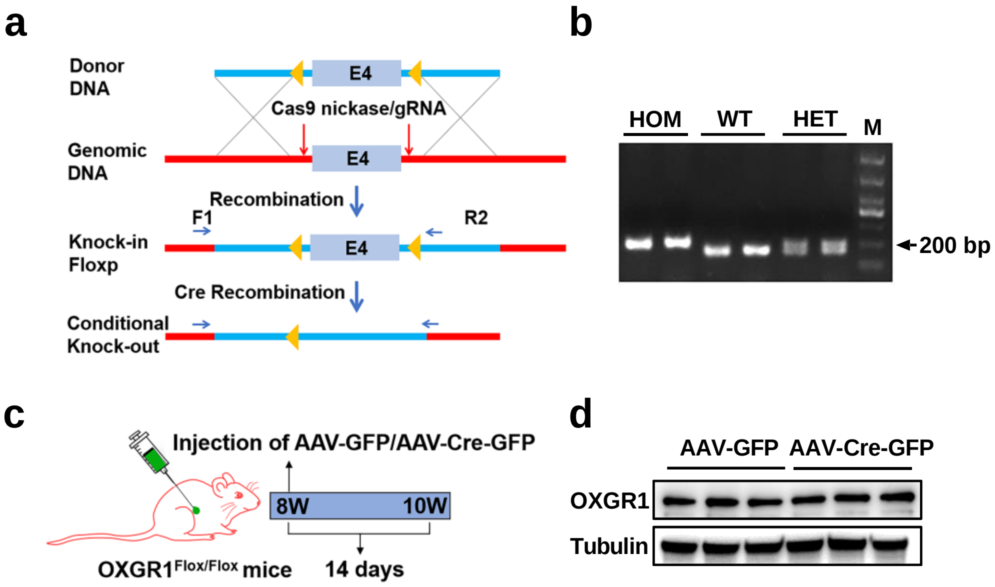

Supplementary Figure. S4

a

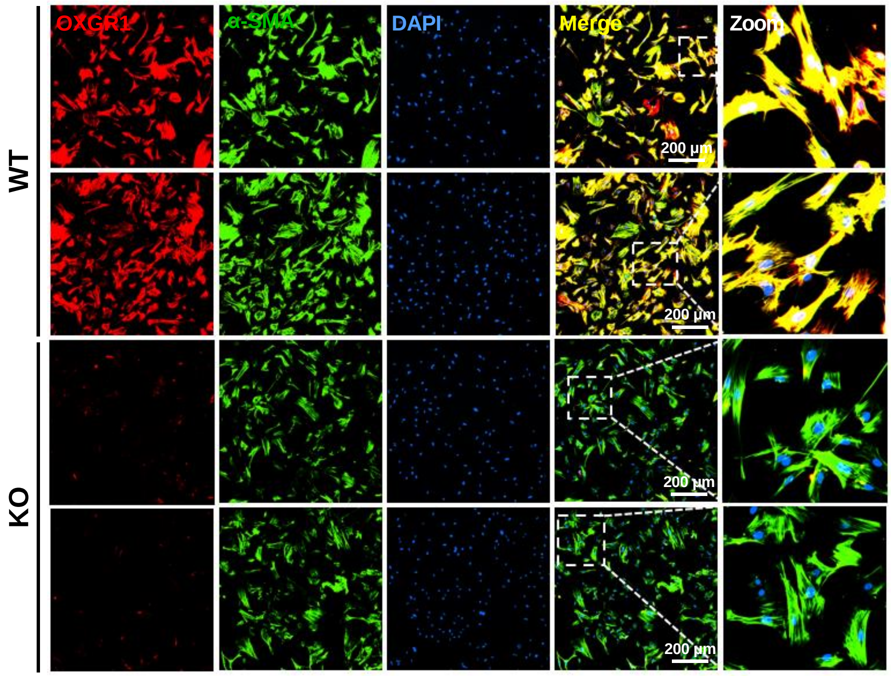

Supplementary Table S1. PCR primer sequences of acid/base transport-related genes.

Gene names	Forward Primer (5'-3')	Reverse Primer (5'-3')
NHE3	GCCTCCGCCTTAGAACATTG	GTCCACCTTTAGCCCCATCT
CAR2	TCACTTTCACTGGGGCTCAT	CCCAAAACAGCCAGTCCATC
CAR4	CAGAGGAGTCGAACAAGGGT	GCCCTCGTTTACCTCGTTTC
CAR14	ACAGAAGGGCCCAGATTTCA	TCCAGCCGAGATTCCCATAC
NBCe1	CCTCTTCCTGGGCACTTACA	TTGGCTTGAACTCACTTGGC
AE2	ACTGCCTTGCTATCACTGGT	ACTGGGCACACTTAGCTTCT
V-ATPase	GCCAATCAATCCCCATGACC	CCCATGGCTGCAAAGACAAT
GAPDH	AGGTCGGTGTGAACGGATTTG	TGTAGACCATGTAGTTGAGGTCA
